# Sterol Intermediates of Cholesterol Biosynthesis Inhibit Hair Growth and Trigger an Innate Immune Response in Cicatricial Alopecia

**DOI:** 10.1371/journal.pone.0038449

**Published:** 2012-06-07

**Authors:** Sreejith P. Panicker, Taneeta Ganguly, Mary Consolo, Vera Price, Paradi Mirmirani, Kord Honda, Pratima Karnik

**Affiliations:** Department of Dermatology, University Hospitals Case Medical Center, Case Western Reserve University, Cleveland, Ohio, United States of America; DRFZ, Germany

## Abstract

Primary cicatricial alopecia (PCA) is a group of inflammatory hair disorders that cause scarring and permanent hair loss. Previous studies have implicated PPARγ, a transcription factor that integrates lipogenic and inflammatory signals, in the pathogenesis of PCA. However, it is unknown what triggers the inflammatory response in these disorders, whether the inflammation is a primary or secondary event in disease pathogenesis, and whether the inflammatory reaction reflects an autoimmune process. In this paper, we show that the cholesterol biosynthetic pathway is impaired in the skin and hair follicles of PCA patients. Treatment of hair follicle cells with BM15766, a cholesterol biosynthesis inhibitor, or 7-dehydrocholesterol (7-DHC), a sterol precursor, stimulates the expression of pro-inflammatory chemokine genes. Painting of mouse skin with 7-DHC or BM15766 inhibits hair growth, causes follicular plugging and induces the infiltration of inflammatory cells into the interfollicular dermis. Our results demonstrate that cholesterologenic changes within hair follicle cells trigger an innate immune response that is associated with the induction of toll-like receptor *(TLR)* and interferon *(IFN)* gene expression, and the recruitment of macrophages that surround the hair follicles and initiate their destruction. These findings reveal a previously unsuspected role for cholesterol precursors in PCA pathogenesis and identify a novel link between sterols and inflammation that may prove transformative in the diagnosis and treatment of these disorders.

## Introduction

Primary cicatricial alopecia (PCA) is a group of rare inflammatory disorders that is characterized by the permanent destruction of the hair follicle. Ultimately, the hair follicle is replaced with fibrous tissue, and progressive and permanent hair loss occurs [1]–[3]. The etiology and pathogenesis of PCA remain unclear, but PCA is currently treated as an inflammatory disorder [2], [4]. The treatment options for PCA are limited and are not very effective in controlling the disease progression. A dearth of information about the underlying molecular pathogenesis has constituted the major obstacle in identifying effective new treatments.

The diagnosis of PCA currently relies upon clinical observation and histological analysis of the inflammatory cell infiltrate in the infundibulum, which is the permanent portion of the hair follicle (HF). Depending on the type of principal inflammatory cell detected during the active phase of the disease, PCA is classified into three categories: *lymphocytic*, including lichen planopilaris (LPP), frontal fibrosing alopecia (FFA) and central centrifugal cicatricial alopecia (CCCA); *neutrophilic,* including folliculitis decalvans (FD) and tufted folliculitis (TF); and *mixed,* including dissecting cellulitis (DC) [5]. In none of the PCA subtypes do we know exactly why hair follicles begin to attract an inflammatory infiltrate. Furthermore, the cellular composition of the inflammatory infiltrate and the nature of its activated state are not well characterized. Thus, it is not surprising that halting or reversing the inflammation in PCA is often difficult.

The failure of the affected follicles to regenerate in PCA is thought to be the result of irreversible changes in the permanent portion of the hair follicles (HF), where the “bulge stem cells” are located [6], [7]. HF bulge stem cells are a unique population of adult stem cells that are multi-potent and play a major role in skin architecture, physiology and wound healing [8], [9]. The permanent loss of hair follicles in PCA is therefore a disastrous event for normal skin function and regeneration. If the HF bulge stem cells are destroyed, there is no possibility for hair follicle regeneration, and permanent hair loss ensues. Thus, PCA offers an excellent model for understanding disease mechanisms where epithelial stem cell populations are targeted by an inflammatory attack.

Our previous studies implicated the peroxisome proliferator-activated receptor gamma (PPARγ), a transcription factor that belongs to the nuclear receptor family, in the pathogenesis of PCA [10]. Nuclear receptors affect transcription through multiple modes of action, including direct activation of genes, ligand-independent repression, ligand-dependent repression, and trans-repression [11]. Members of the nuclear receptor family, including PPARγ, modulate the expression of lipogenic and inflammatory genes and have emerged as important regulators of metabolic and inflammatory signaling. We previously reported a loss of PPARγ signaling in the PCA subtype LPP. Indeed, the deletion of PPARγ in K15-bulge stem cells in mutant mice results in an LPP-like skin phenotype with progressive hair loss, perifollicular inflammation and scarring alopecia [10]. Gene expression profiling of the skin from PPARγ knockout mice and from scalp tissue in patients affected with LPP consistently showed decreased expression of PPARγ-regulated lipid metabolic genes and increased expression of inflammatory genes [10]. Although PPARγ has emerged as an important regulator of lipogenic and inflammatory genes [11], the precise link between specific lipid metabolic pathways and inflammation in the skin and skin appendages is not fully understood [12].

In this manuscript, we demonstrate that the cholesterol biosynthesis pathway is altered in all subtypes of PCA. We further show that changes in cholesterol biosynthesis within hair follicle cells trigger a pro-inflammatory response and induce the recruitment of innate immune cells that initiate the destruction of hair follicles in mouse skin and in PCA. Our results reveal a previously unidentified role for cholesterol precursors in PCA pathogenesis and identify a novel link between sterols and inflammation that may prove transformative in the diagnosis and treatment of these disorders.

## Results

### Altered Cholesterologenic Program in PCA

**Figure 1:**
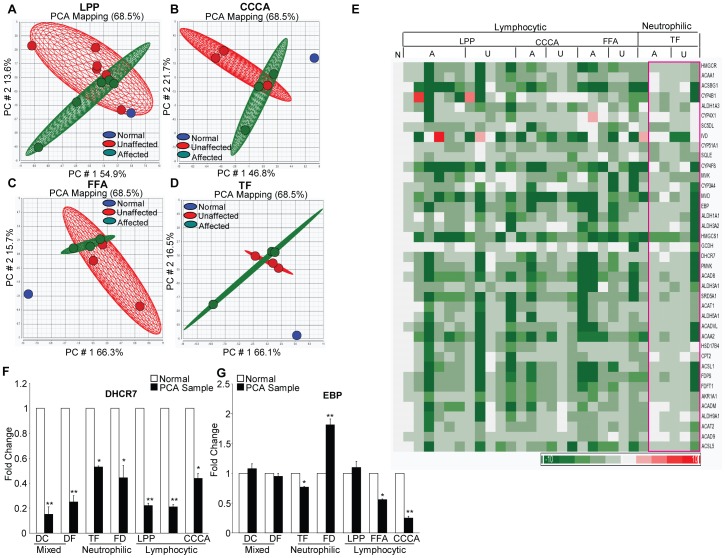
Decreased expression of genes related to cholesterol biosynthesis and lipogenesis in PCA. Principal component analysis of microarray data (downregulated genes) from lymphocytic (1A–1C) and neutrophilic (1D) cicatricial alopecia was performed with the Partek Genomics Suite. The results of the analysis for LPP are shown in 1A, for CCCA in 1B, for FFA in 1C and for TF in 1D. The horizontal axis corresponds to principal component 1 (PC1), the vertical axis corresponds to PC2 and the depth axis corresponds to PC3. The points are colored by group status: blue represents normal samples (pooled), green represents unaffected samples and red represents affected cicatricial alopecia samples. The clustering of data by samples suggests similarities in gene expression profiles. Unaffected and affected samples are clustered together in each subtype, which suggests that the expression profiles of genes involved in cholesterol biosynthesis and lipogenesis are not significantly different among these samples. The normal scalp tissue was significantly different from the unaffected and affected scalp samples from patients with LPP, CCCA, FFA and TF. (E) Heat map of the 39 most significantly downregulated genes in patients with LPP (6 affected and 5 unaffected scalp samples) CCCA, FFA and TF (3 affected and 3 unaffected scalp samples each). The majority of the genes participated in cholesterol biosynthesis. The color bar below indicates the level of expression. (F) Real-time PCR validation of *DHCR7* gene expression in normal skin and in the PCA subtypes LPP, CCCA, FFA, TF and DF (*p<0.05, **p<0.01). Compared with normal tissue, *DHCR7* expression was significantly decreased in all PCA samples. The unpaired *t-*test was used for statistical analysis. (G) Real-time PCR validation of *EBP* gene expression in skin from normal controls and patients with the PCA subtypes LPP, CCCA, FFA, TF and DF (*p<0.05, **p<0.01). *EBP* expression was significantly decreased in the PCA subtypes TF, FFA and CCCA but not in DC, FD or LPP. The unpaired *t-*test was used for statistical analysis. See also Figure S1 and Table S1.

To identify the pathways underlying PCA pathogenesis, we analyzed the gene expression profiles of paired unaffected (non-lesional) and affected (lesional) scalp tissues from 12 lymphocytic (LPP, CCCA, FFA) and 3 neutrophilic (TF) PCA patients. These tissues were compared to normal scalp tissue (N = 10 pooled) from healthy individuals using Affymetrix microarrays. Principal component analysis based on all downregulated genes and all samples revealed a 68.5% variation between diseased and normal samples in the first two principal components (Figure 1A–1D). The unaffected (green ovoid) and affected (red ovoid) samples in each PCA subset, including LPP (Figure 1A), CCCA (Figure 1B), FFA (Figure 1C) and TF (Figure 1D), formed distinct groups and showed a partial overlap. In contrast, the normal controls (N = 10 pooled, blue ovoid) were well separated from both the unaffected and the affected samples from the patients (Figure 1A–1D). In LPP (Figure 1A), the normal controls lie within the plane of unaffected samples. This is due to the smaller number of gene expression changes in unaffected LPP compared to normal samples. These analyses indicate a distinct gene expression profile of unaffected scalp skin in all PCA subtypes. This gene expression profile has some similarities to that of the affected scalp skin from the same patients, but is markedly different from the profile of normal healthy controls.

The most extensively downregulated gene clusters in PCA are shown as a heat map (Figure 1E) and consist of 39 genes dedicated to lipid metabolism. The majority of the altered transcripts belonged to the cholesterol biosynthesis pathway. The remaining transcripts were associated with a few predominant pathways, including fatty acid metabolism, the aldehyde dehydrogenase-related pathway for the oxidation of endogenous and xenobiotic aldehydes and pathways involving the cytochrome P450 superfamily of enzymes, which catalyze many reactions in the synthesis of cholesterol, steroids and other lipids. The downregulation of genes involved in cholesterol biosynthesis in both unaffected and affected tissue from patients with PCA suggests that these genes are involved in the early changes in PCA pathogenesis.

The decreased expression of genes related to cholesterol biosynthesis in PCA was confirmed in an independent set of PCA samples by microarray analysis (Table S1) and real-time PCR (Figure 1E). As shown in Table S1, the number of cholesterol biosynthesis genes affected and the extent to which they are downregulated differ in the different PCA subtypes. The genes that encode *DHCR7, HMGCS1* and *SC5DL* are downregulated in all subtypes; *ACAT2, FDPS* and *PMVK* are downregulated in lymphocytic but not in neutrophilic PCA; *MVK* is downregulated only in neutrophilic PCA; and *MVD* is downregulated only in FFA (Gene names are included in Glossary S1). We validated the microarray data using the real-time PCR with primers specific for the two cholesterol biosynthesis genes, *DHCR7* (Figure 1F) and *EBP* (Figure 1G). *DHCR7* was significantly downregulated in both the lymphocytic and neutrophilic types of PCA, as shown in Figure 1F. In contrast, the expression of *EBP* differed in the different subtypes. *EBP* was significantly downregulated in the lymphocytic PCA subtypes CCCA and FFA. However, the expression of *EBP* did not significantly change in neutrophilic PCA. These data suggest that, although cholesterol biosynthesis is decreased in all PCA subtypes, different genes in this biosynthetic pathway are affected in the different subtypes (Table S1 & Figure 1F–1G).

**Figure 2:**
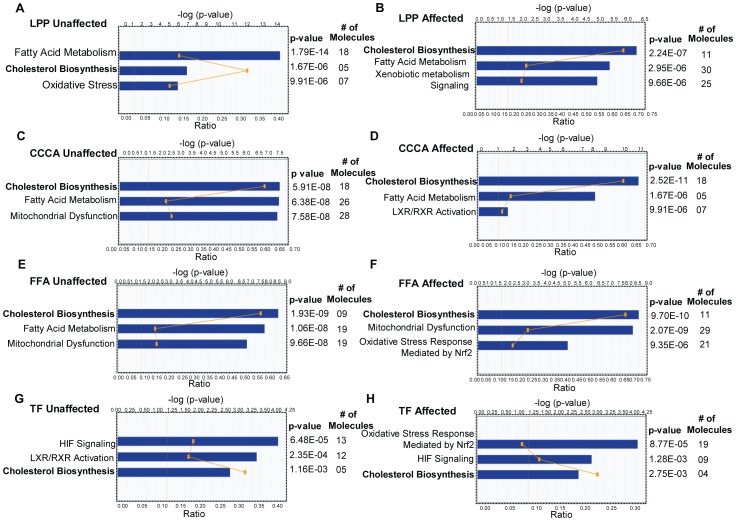
Ingenuity Pathways Analysis of the top toxic pathways in cicatricial alopecia. IPA-Tox®, a data analysis capability tool within the Ingenuity Pathways Analysis, was used to analyze the microarray data and to determine the toxicity associated with the observed gene expression changes in PCA. The figure shows the top toxicity lists (Toxlists) associated with gene expression changes in samples from unaffected and affected scalp areas in patients with LPP, CCCA, FFA and TF. Cholesterol biosynthesis appears to be the most significant toxicity-related pathway associated with the lymphocytic PCA subtypes.

To determine the biological relevance of the gene expression changes in PCA, we analyzed the microarray data with the Ingenuity Pathway Analysis (IPA) software (www.ingenuity.com, Ingenuity Systems Inc., Redwood City, CA, USA). IPA provides the ability to map differentially expressed genes to fixed canonical pathways and Toxlists. IPA-Tox® is a data analysis capability within IPA that identifies biological mechanisms that are related to toxicity (Toxlists) (on a molecular, cellular, and biochemical level). Figure 2 summarizes the most significant Toxlists associated with the gene expression changes observed in unaffected and affected tissues from patients with LPP, CCCA, FFA and TF. Cholesterol biosynthesis appears at the top of the Toxlist (Figure 2A–2F) suggesting that it is the most significant toxic pathway associated with the lymphocytic PCA subtypes. The other significant pathways in lymphocytic PCA include fatty acid metabolism, oxidative stress response, mitochondrial dysfunction and LXR/RXR activation. In contrast, the oxidative stress response and hypoxia-inducible factor (HIF) signaling are the most significant toxic pathways associated with neutrophilic PCA (Figure 2G–2H). As shown in Figure 2A–2F, although the Toxlists are remarkably similar, there are also distinct differences between the different subtypes of lymphocytic PCA.

Together, these observations suggest that cholesterol biosynthesis is significantly decreased in PCA and is the most significant toxic pathway associated with the lymphocytic PCA. The cholesterol biosynthesis pathway is also affected in neutrophilic PCA, albeit to a lesser extent. We have therefore identified a “pre-PCA” gene expression signature in uninvolved tissue from PCA patients. This signature is comprised of downregulated cholesterol biosynthesis genes that may represent the earliest changes in disease pathogenesis.

### Increased Expression of Adaptive and Innate Immune Genes in PCA

**Figure 3:**
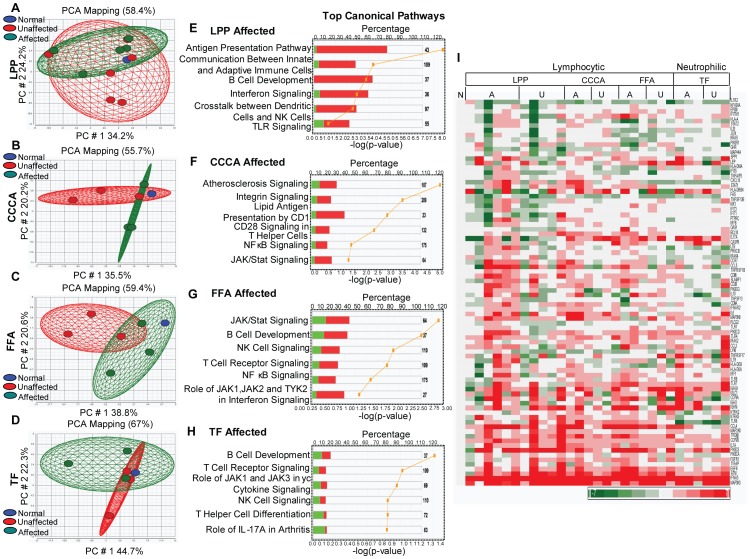
Increased expression of immune and inflammatory genes in PCA. The principal component analysis results for upregulated genes from lymphocytic (A) LPP, (B) CCCA, (C) FFA and (D) neutrophilic (TF) cicatricial alopecia are shown. The normal and unaffected samples are clustered together in each subtype, which suggests that the expression of immune and inflammatory genes is not significantly different among these samples. Most samples from affected scalp areas in patients with PCA are clustered separately from normal controls and from samples of unaffected scalp skin from PCA patients, which suggest that the expression of these genes differs between affected and unaffected samples. The top canonical pathways from gene expression profiles in patients with (E) LPP, (F) CCCA, (G) FFA and (H) TF are shown. Red represents upregulated and green represents downregulated genes in these pathways. The yellow graph line in E, F, G and H represents –log (p values). (I) Heat map of the most significantly altered immune and inflammatory genes in LPP (6 affected and 5 unaffected samples), CCCA, FFA and TF (3 affected and 3 unaffected samples each) is shown. The color bar below indicates the level of expression.

The comparison of gene expression profiles of PCA scalp skin with healthy control skin by microarray analysis revealed that the most prominently upregulated genes are those involved in the immune and inflammatory responses (Figure 3). A principal component analysis based on all upregulated genes in all samples revealed a near-complete separation of the affected tissue from the unaffected and normal tissue in all PCA subtypes (Figures 3A–3D). As shown in Figures 3A–3D, the unaffected tissue from PCA patients (green ovoid) lies on the same plane as the pooled normal control tissue (N = 10 pooled, represented by the blue ovoid). These data suggest that the expression of inflammatory genes is altered in the affected tissue of PCA patients, and there is no significant difference in the expression of inflammatory genes between the unaffected tissue of PCA patients and normal controls. The differential expression of immune response gene clusters in the affected, but not unaffected, scalp skin of PCA patients suggests that the inflammatory changes occur in the “active disease” and do not represent the earliest changes in PCA pathogenesis.

A pathway analysis of the microarray data from affected scalp tissue revealed that the innate and the adaptive immune pathways are both upregulated in PCA (Figure 3E–3H). In LPP (Figure 3E), the upregulated inflammatory pathways include the antigen presentation pathway, communication between adaptive and innate immune cells, B-cell development, interferon signaling, crosstalk between dendritic and NK cells and Toll-like receptor (TLR) signaling. The upregulated inflammatory pathways in CCCA (Figure 3F) include atherosclerosis and integrin signaling, lipid antigen presentation by CD1, CD28 signaling in T helper cells and NFkB and JAK/Stat signaling. In FFA (Figure 3G), the major upregulated pathways include JAK/Stat signaling, B-cell development, NK cell signaling, T-cell receptor and NFkB signaling. The upregulated pathways in neutrophilic PCA (TF) (Figure 3H) include B-cell development, T-cell receptor signaling, NK cell signaling, T helper cell differentiation and IL-17A.

**Figure 4:**
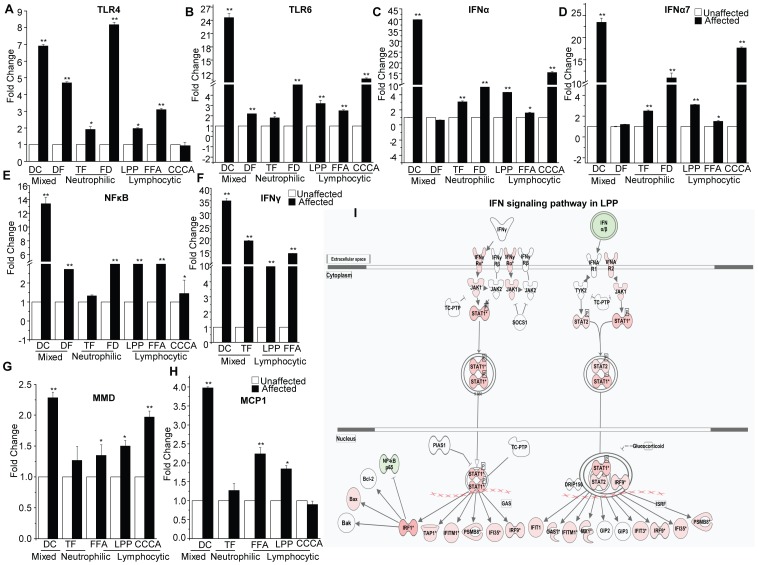
Innate immune genes are upregulated in PCA. Real-time PCR validation of (A) *TLR4,* (B) *TLR6,* (C) *IFNα*, (D) *IFNα7,* (E) *NFkB,* (F) *IFNγ*, (G) *MMD* and (H) *MCP1* in mixed (DC, DF), neutrophilic (TF, FD) and lymphocytic (FFA, LPP, CCCA) PCA. These genes are significantly upregulated in affected tissue compared to unaffected tissue from the same patients (*p<0.05, **p<0.01). The unpaired *t-*test was used for statistical analysis. Differences in the pattern of expression of these genes were observed in the different PCA subtypes. (I) IPA identified the interferon signaling pathway or the “interferon-responsive signature” in the gene expression profiles of LPP. The intensity of the node color red indicates the degree of upregulation, and the intensity of the color green indicates the degree of downregulation. Genes shown as uncolored nodes were not identified as differentially expressed in our experiment and were integrated into the computationally generated networks based on the evidence stored in the IPA knowledge base, which indicated a relevance to this network. The node shapes denote enzymes, phosphatases, kinases, peptidases, transmembrane receptors, cytokines, transporters, translation factors, nuclear receptors and transcription factors. The interferon target genes *IRF1, IRF8, IFNA5, IFNAR2, IFIT3*, *IFITM1, MX1, OAS1* and *IFI35* are significantly upregulated in LPP. See also Table S2.

The predominantly upregulated inflammatory genes in PCA are shown as a heat map (Figure 3I). Various genes were identified in the “immune cluster,” including genes whose products control the innate immune responses, such as the interleukin/Toll-like receptor superfamily, interferon inducible proteins, and monocyte/macrophage related proteins. Genes whose products are required for the adaptive immune response, such as chemokine/cytokine family members, T and B cell activation and survival genes, genes encoding MAP kinases, members of the tumor necrosis factor superfamily and the major histocompatibility complex class I and class II genes, were also overexpressed in PCA. The microarray data were validated by performing real-time PCR for *TLR4, TLR6, IFNα, IFNα7, NFkB* and *IFNγ* in tissues from patients with lymphocytic and neutrophilic PCA. As shown in Figure 4A–4H, real-time PCR with target-gene-specific primers confirmed that Toll-like receptor *(TLR4, TLR6)*, interferon *(IFNα, IFNα7, IFNγ)*, pro-inflammatory cytokine *(NFkB)* and macrophage activation factor *(MMD, MCP1)* genes are all significantly upregulated in affected PCA tissue compared with unaffected tissue from the same patients.

Intriguingly, the IFN signaling pathway and its target genes that serve as a link between innate and adaptive immunity were upregulated in affected but not unaffected tissue from patients with all PCA subtypes (lymphocytic, neutrophilic and mixed), as shown in Figures 4C, 4D, 4F & 4I. Interferons can modulate the expression of several hundred genes encoding proteins that participate in the antiviral defense, inflammation, adaptive immunity and angiogenesis. The result is a characteristic pattern of mRNA expression known as the "interferon signature." The upregulation of interferon signature genes in LPP was confirmed using IPA (Figure 4I, Table S2). The interferon-responsive signature genes upregulated in LPP include type II interferon *(IFNγ)*-responsive genes *(IRF1, TAP1, IFITM1, PSMB8, IFI35*, and *IRF9)* and type I interferon *(IFNα)*-responsive genes *(IFIT1, OAS1, IFITM1, MX1, IFIT3, IRF9, IFI35*, and *PSMB8)*, as shown in Figure 4I and Table S2.

These data provide evidence for the involvement of the innate and adaptive immune responses in the pathogenesis of PCA.

### Sterol Intermediates of Cholesterol Biosynthesis Induce a Pro-inflammatory Response in Hair Follicle Cells *in vitro*

Given the reciprocal expression pattern for genes related to cholesterol biosynthesis (downregulated) and inflammation (upregulated) in PCA, we hypothesized that a mechanistic link exists between these diverse signaling pathways in hair follicles. To test this hypothesis and to determine the physiological relevance of decreased cholesterol biosynthesis in PCA, we studied the effects of the cholesterol biosynthesis inhibitor BM15766 (a pharmacological inhibitor of the enzyme DHCR7), and 7-dehydrocholesterol (7-DHC) (a cholesterol precursor) on human hair follicle outer root sheath (HHFORS) cells in vitro. The cholesterol biosynthesis pathway involves ≥20 enzymatic reactions. As shown in Figure S1, the last step in cholesterol biosynthesis is the conversion of 7-DHC to cholesterol by 7-dehydrocholesterol reductase (DHCR7). We selected the DHCR7 inhibitor BM15766 and the cholesterol precursor 7-DHC for our studies because our data showed that the expression of DHCR7 was consistently and significantly decreased in all tested PCA samples (Figure 1F & Table S1). We suspected that decreased expression of DHCR7 would cause an accumulation of the cholesterol biosynthesis intermediate 7-DHC in hair follicle cells.

**Figure 5:**
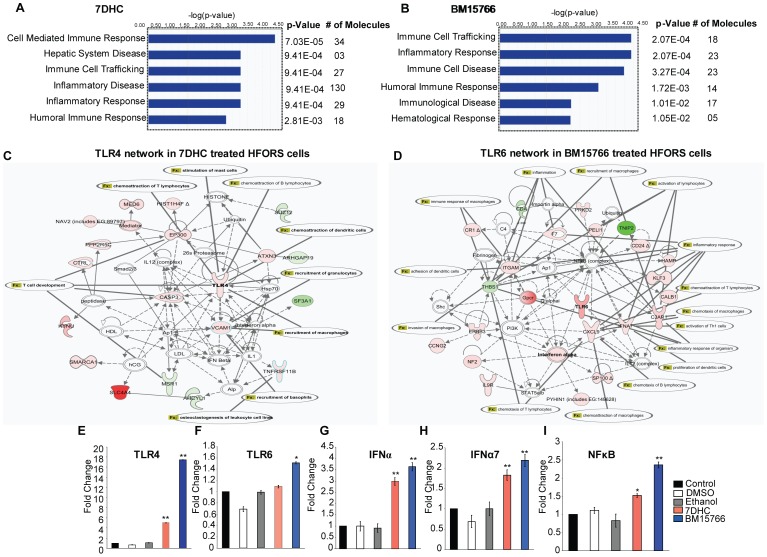
Sterol intermediates of cholesterol biosynthesis trigger an inflammatory response in hair follicle cells. Inflammatory and immune responses were the top biological functions affected by treatment of HHFORS cells with (A) 7-DHC or (B) BM15766. The most significant biological functions affected, their p values and the number of differentially expressed genes (molecules) after each treatment were identified using IPA. The difference between vehicle and 7-DHC or BM15766 treatments was defined as significant if a 1.5-fold or greater difference in the average hybridization signal intensity with a p<0.05 using a two-tailed unpaired *t-*test was observed. Predicted interaction networks in hair follicle cells after treatment with (C) 7-DHC and (D) BM15766 are shown. The *TLR4* gene network was activated after 7-DHC treatment, and the *TLR6* network was activated after BM15766 treatment. Solid lines denote a direct relationship, and dotted lines denote an indirect relationship between two genes in the network. A red node denotes an upregulated gene, and a green node denotes a downregulated gene, with the difference in intensity reflecting the degree of change in the expression of differentially expressed genes in our dataset. The inflammatory functions and disease (fx) associated with each *TLR* network were determined using IPA. See also Table S3. The real-time PCR validation of (E) *TLR4*, (F) *TLR6*, (G) *IFNα*, (H) *IFNα7* and (I) *NFkB* gene expression in 7DHC- and BM15766-treated hair follicle cells (*p<0.05, **p<0.01) is shown. The unpaired *t-*test was used for statistical analysis.

Treatment of HHFORS cells with 7-DHC or BM15766 induced a pro-inflammatory response, as determined by global gene expression profiling and real-time PCR (Figure 5). An IPA analysis of the microarray data for 7-DHC- or BM15766-treated HHFORS cells revealed a significant increase in the expression of inflammatory genes (Figures 5A & 5B). We found that the most significant pathways affected in 7-DHC-treated cells were the cell mediated-immune response, immune cell trafficking, inflammatory disease, the inflammatory response and the humoral immune response (Figure 5A). The most significant pathways affected in BM15766-treated cells were immune cell trafficking, the inflammatory response, immune cell disease, immunological disease and the hematological response (Figure 5B). Intriguingly, the most significant predicted networks identified by IPA analysis included the *TLR4* network in 7-DHC-treated cells (Figure 5C, Table S3) and the *TLR6* network (Figure 5D, Table S3) in BM15766-treated cells.

Real-time PCR confirmed that the expression of innate immune and pro-inflammatory genes significantly increased in 7-DHC- and BM15766-treated HHFORS cells compared with untreated or vehicle-treated controls (Figure 5E–5I). The expression of *TLR4* was significantly increased in HHFORS cells treated with 7-DHC (∼5-fold, p<0.05) or BM15766 (∼18-fold, p<0.05), as shown in Figure 5E. In contrast, *TLR6* was specifically upregulated in cells treated with BM15766 (∼1.6-fold, p<0.01), but not in cells treated with 7-DHC. After treatment with 7-DHC or BM15766, *IFN-α* (∼3-fold, p<0.05 and ∼3.5-fold, p<0.05; Figure 5G), *IFN-α7* (∼3.5-fold, p<0.05 and ∼4-fold, p<0.05; Figure 5H) and *NFkB* (∼1.7-fold, p<0.05 and ∼2.25-fold, p<0.05; Figure 5I) were all significantly upregulated in HHFORS cells, respectively. In addition, real-time PCR showed that the expression of the TGFβ1 gene was significantly upregulated in hair follicle cells after treatment with BM15766 (Figure S2). These data suggest that the inhibition of endogenous cholesterol biosynthesis or the accumulation of cholesterol precursors induces a pro-inflammatory response in human hair follicle cells in vitro.

### Sterol Intermediates of Cholesterol Biosynthesis Inhibit Hair Growth and Trigger an Innate Immune Response in Mouse Skin

**Figure 6:**
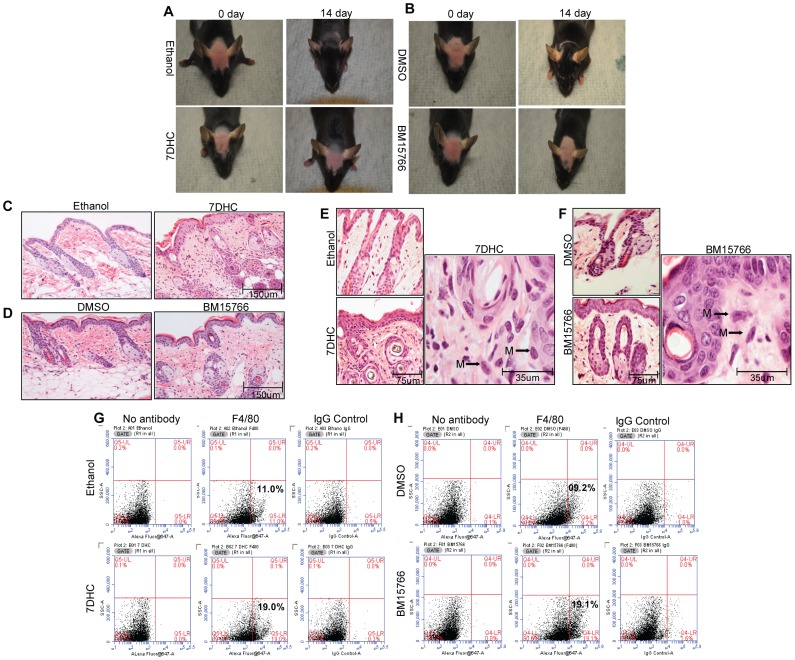
Sterol intermediates of cholesterol biosynthesis inhibit hair growth and activate the inflammatory response in C57BL/6J mice. (A) Topical 7-DHC and (B) BM15766 treatment inhibited hair growth compared with vehicle treatment (ethanol or DMSO). Hair growth was restored in vehicle-treated but not in 7-DHC and BM15766 treated mice. (C, D) Histology of mouse skin treated with ethanol, DMSO, 7-DHC and BM15766 (20X). H&E staining of vehicle-treated mice showed normal hair follicles and sebaceous glands. In contrast, H&E staining of mice treated with 7-DHC and BM15766 showed hyperkeratosis as well as dystrophic hair follicles and sebaceous glands. Scale bar = 50 µm. (E, F) H&E staining showed peri-follicular and inter-follicular inflammation in mouse skin that had been treated with 7-DHC (40×). At higher magnification (100×), infiltration of tissue histiocytes is observed. (G, F) A higher percentage of F4/80 cells was present in mouse skin treated with 7-DHC (Q5-LR) and BM15766 (Q4-LR) than in the vehicle-treated mouse skin. See also Figures S3 and S4.

We next determined whether BM15766 and 7-DHC have the same effects on mouse skin in vivo (Figure 6). Mouse hair cycle stages are naturally synchronized in individual mice. However, animals of the same age and from the same litter can exhibit heterogeneity with respect to hair follicle cycling. Therefore, mice in the telogen phase of hair growth cycle (7 weeks old) were depilated to synchronize their hair cycle stages and painted every day for 14 days with 7-DHC (a cholesterol precursor; see Figure 6A), BM15766 (an inhibitor of cholesterol biosynthesis; see Figure 6B) or vehicle (ethanol or DMSO; see Figures 6A & 6B). The mice were then monitored for hair re-growth. Histological changes were monitored by H&E staining of paraffin-mounted mouse skin sections. At the end of 14 days, hair growth was fully restored in the vehicle-treated mice. Ethanol-treated mice are shown in Figure 6A and DMSO-treated mice in Figure 6B. In stark contrast, the hair follicles in mice treated with 7-DHC (Figure 6A) or BM15766 (Figure 6B) did not re-grow. We therefore determined whether BM15766 or 7-DHC had an effect on catagen induction or on hair follicle stem cells. As shown in Figure S2, real-time PCR showed that the expression of the TGFβ1 gene was significantly increased in mouse skin tissue after treatment with BM15766. In addition, the expression of SOX9 was significantly decreased both in HHFORS cells in culture and in mouse skin after treatment with 7-DHC and BM15766 (Figure S2).

H&E staining of mouse skin treated with 7-DHC or BM15766 showed epidermal thickening, follicular plugging and an increased number of histiocytes in the dermis (Figure 6C & 6D). Tissue macrophages in the inter-follicular dermis and surrounding hair follicles were detected at higher magnification (100X) in H&E stained skin sections from mice treated with 7-DHC or BM15766 (Figure 6E & 6F).

Macrophages are crucial initiators and regulators of the innate and adaptive host defenses in the skin. Therefore, to corroborate the H&E data, we stained the mouse skin sections with antibodies to F4/80, which is a transmembrane protein present on the surface of mouse macrophages. As shown in Figure S3, F4/80-positive cells were observed in mice treated with 7-DHC and BM15766 but not in vehicle-treated controls. Flow cytometry analysis also revealed a significantly increased number of macrophages (F4/80+ cells) in mouse skin that had been treated with 7-DHC (8% increase; see Figure 6G) or with BM15766 (9% increase; see Figure 6H) compared with vehicle alone (Figure 6G & 6H). To determine if macrophages were present in the tissue of patients with PCA, we stained scalp skin sections from patients with lymphocytic and neutrophilic PCA with antibodies to CD68, a 110-kD transmembrane glycoprotein that is highly expressed in human monocytes, including tissue macrophages. Figure S4 shows a microphotograph of CD68+ cells in the lymphocytic (LPP, CCCA, and FFA) and neutrophilic (TF) subtypes of PCA. These results suggest a role for macrophages in the pathogenesis of PCA.

**Figure 7:**
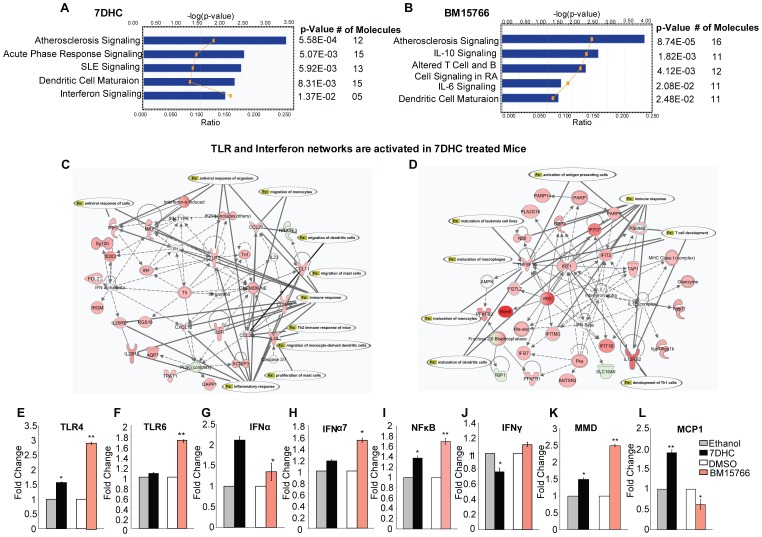
Inflammatory pathways and networks activated in C57BL/6J mouse skin after topical treatment with 7-DHC and BM15766. (A) The most significant signaling pathways altered by 7-DHC treatment participated in the inflammatory and immune responses and were identified using IPA. Fisher’s exact test was used to calculate p values to determine the probability that the association between the genes in the dataset and the pathway could be explained by chance alone. The yellow line indicates the threshold of significance (p<0.05) and represents the ratio of the number of molecules from the data set that map to the pathway to the total number of molecules that map to the pathway. (B) The top differentially regulated pathways in BM15766-treated mouse skin. The majority of the upregulated pathways participated in the inflammatory and immune responses. (C, D) The top two predicted networks in 7DHC-treated mouse skin, determined using IPA. The *TLR4* and *IFN* gene networks are significantly upregulated by 7-DHC. Solid lines denote direct relationships between genes. Dotted lines denote an indirect relationship between two genes. A red node denotes an upregulated gene, and a green node denotes a downregulated gene. See also Table S4. Real-time PCR validation of (E) *TLR4*, (F) *TLR6*, (G) *IFNα*, (H) *IFNα7*, (I) *NFkB*, (J) *IFNγ,* (K) *MMD* and (L) *MCP1* gene expression in mouse skin treated with 7-DHC or BM15766 compared with vehicle-treated (ethanol or DMSO) controls (n = 3; *p<0.05, **p<0.01). The unpaired *t-*test was used for the statistical analysis. Treatment with 7-DHC and BM15766 can induce the expression of some or all of these genes.

To elucidate the mechanisms and signaling pathways underlying our findings, we performed gene expression profiling and IPA analysis to compare mouse skin that had been treated with 7-DHC and BM15766 with untreated controls. The inflammatory and immune pathways that were upregulated in 7-DHC-treated mouse skin included acute phase response signaling, SLE signaling, dendritic cell maturation and interferon signaling (Figure 7A). Pathways that were upregulated in BM15766-treated mouse skin included IL-10 signaling, T- and B-cell signaling in RA, IL-6 signaling and dendritic cell maturation (Figure 7B). As observed in vitro, we identified upregulation of TLR (Figure 7C, Table S4) and interferon signaling networks (Figure 7D, Table S4) in 7-DHC - treated mouse skin.

As shown in Figures 7E-L, real-time PCR confirmed the microarray data showing that innate immune response genes, specifically *TLR* and *IFN* genes are significantly upregulated with sterol treatment. We observed that *TLR4* (∼3-fold, p<0.05), *TLR6* (∼3-fold, p<0.05), *IFNα1* (∼1.4-fold, p<0.01), *IFNα7* (∼1.5-fold, p<0.01) and *NFkB* (∼1.7-fold, p<0.05) were significantly upregulated in BM15766-painted mouse skin. In contrast, *TLR4* (∼1.6-fold, p<0.01) and *IFNα1* (∼2.2-fold, p<0.05) were significantly upregulated in 7-DHC-painted mouse skin.

## Discussion

The central finding of this study is that cholesterologenic changes within hair follicle cells trigger an innate immune response that leads to the induction of *TLR* and *IFN* gene expression and the recruitment of macrophages that surround the hair follicles and initiate their destruction.

Although PCA is a group of inflammatory hair disorders, several unanswered but critical questions remain regarding the nature and role of the inflammatory infiltrate in the natural history of this disease. Several questions remain unanswered, including whether the inflammatory reaction represents a primary or secondary event in the disease pathogenesis, whether the inflammation is triggered by changes within hair follicles or by exogenous factors (e.g., pathogens) and how much of the inflammatory reaction reflects an autoimmune process. This study provides a framework for addressing these complex issues.

Our data suggest that cholesterol precursors generated within hair follicle cells trigger the initial inflammatory response and induce the recruitment of tissue macrophages in mouse skin and in PCA. These conclusions are based on several observations. Using a global gene expression analysis, we first showed that the expression of genes related to cholesterol biosynthesis is significantly decreased in unaffected and affected scalp tissue from PCA patients, which suggests that these expression changes are early events in the pathogenesis of this disease. We further demonstrated that innate and adaptive immune genes and pathways are upregulated in lesional tissue, and there is a reciprocal expression pattern of cholesterologenic and inflammatory gene expression in PCA. Our data suggest that changes in expression of cholesterol biosynthesis genes are a hallmark of all PCA subtypes and underlie the pathogenesis of this group of alopecia. Our previous studies [10] showed that the hair follicles and sebaceous glands of PPARγ knockout mice (a mouse model of scarring alopecia) have decreased expression of lipid metabolic genes and increased expression of inflammatory genes suggesting that these changes are caused by loss of PPARγ signaling [10].

Based on our current observation that the cholesterol biosynthesis pathway is decreased in all PCA subtypes, we explored the possibility of a mechanistic link between cholesterol biosynthesis and the inflammatory response in PCA. We treated hair follicle cells in vitro with BM15766, a cholesterol biosynthesis inhibitor, or 7-DHC, a cholesterol precursor, and we observed that these substances induced a pro-inflammatory response with increased expression of *TLR* and *IFN* genes. Our findings that the *TLR4* gene network is activated after treatment with 7-DHC and that the *TLR6* network is activated after treatment with BM15766 (causes accumulation of 7-DHC & 8-DHC) may help to explain the pathogenesis of PCA. Our data suggest that different sterols induce diverse chemokine and cytokine profiles. These observations are relevant to PCA because different sterols generated in the subtypes of PCA may contribute to the clinical and histological differences among the different disease entities.

We next asked whether the association between cholesterol biosynthesis and the inflammatory response observed in vitro could be reproduced upon treatment with BM15766 or 7-DHC in vivo. Intriguingly, the expression of TGFβ1, a catagen inducer [13] was significantly upregulated both in HHFORS cells and in mouse skin after treatment with BM15766. In addition, the expression of SOX9, a hair follicle bulge stem cell marker [14] was significantly downregulated in both human hair follicle cells and in mouse skin upon treatment with 7-DHC and BM15766. A histological analysis of the mouse skin that had been treated with BM15766 and 7-DHC showed follicular plugging, epidermal thickening and an inflammatory infiltrate in the inter-follicular dermis. These data suggest that inhibition of cholesterol biosynthesis with BM15766 or treatment with the intermediate 7-DHC inhibits hair growth and arrests the hair growth cycle in mouse skin. F4/80 staining of the inflammatory infiltrate showed that macrophages are the first inflammatory cells to appear upon treatment of mouse skin with BM15766 or 7-DHC. Intriguingly, we observed the CD68+ staining of scalp tissue from patients with neutrophilic and lymphocytic PCA, which suggests a role for macrophages in the pathogenesis of PCA.

We further observed that BM15766 and 7-DHC-treated mice express prominent transcriptional signatures related to *TLR* and *IFN* signaling in their skin. TLRs are inflammatory molecules that bridge the innate and adaptive immune systems in humans and activate multiple inflammatory pathways [13]–[15]. TLRs coordinate the systemic defense against pathogens and may recognize self-lipids, proteins and endogenous nucleic acids [16]. Data originating predominantly from animal models of autoimmune disease and anecdotal data from human patients suggest that the inappropriate activation of TLR pathways by endogenous or exogenous ligands may lead to the initiation and/or perpetuation of autoimmune responses and tissue injury. TLRs in non-immune cells have been implicated in multiple autoimmune and inflammatory diseases, including Hashimoto’s thyroiditis (TLR3 in thyrocytes) [17], colitis (TLR4 in intestinal epithelial cells [18] and type 1 diabetes (TLR3 in pancreatic ß-cells) [19]. In each case, the pathological expression of the TLR in non-immune cells is associated with an autoimmune/inflammatory disease. Therefore, the induction of TLRs by 7-DHC and BM15766 in hair follicle cells in vitro and in mouse skin in vivo in the present study is a novel observation.

We demonstrated that the inhibition of cholesterol biosynthesis or treatment with cholesterol precursors in hair follicle cells induces the expression of IFN and its target genes in mouse skin and in tissue from patients with PCA. Intriguingly, the gene expression profiling of tissue from patients with LPP, CCCA, FFA and TF revealed the upregulation of interferon genes in all subtypes. However, an “interferon-responsive signature” of interferon target genes was clearly detected in LPP. Our data show for the first time that LPP has an gene expression signature that is related to the “interferon response” that is commonly observed in autoimmune diseases [20]. Analyses of additional samples may be necessary to identify similar signatures in other PCA subtypes. The “interferon response signature” is emerging as a common diagnostic feature of diverse autoimmune diseases, such as type 1 diabetes (T1D) [21], autoimmune thyroid disease [22], systemic lupus erythematosus (SLE) [23], rheumatoid arthritis (RA) [24], scleroderma [25], Sjogren’s syndrome [26], dermatomyositis [27] and psoriasis [28], as well as animal models of autoimmune diseases. Our data show a direct link between sterols and the activation of the TLR and IFN signaling pathways in mouse skin and in LPP. The TLR and IFN gene expression signatures in PCA are similar to those observed in many autoimmune diseases. However, it remains to be determined if PCA are indeed autoimmune diseases.

In conclusion, our data suggest that cholesterologenic changes are the primary events in the pathogenesis of PCA and trigger an inflammatory response in human hair follicle cells and in mouse skin. One mechanism by which sterol precursors might induce the expression of TLR and IFN genes could be via the activation of liver X receptors (LXR). LXRs are nuclear receptors activated by sterol intermediates of cholesterol biosynthesis [29]. They function as cholesterol sensors and regulators [30] and also control transcriptional programs involved in the inflammatory response. Although LXR has been reported to have anti-inflammatory effects [31], [32], LXR-dependent gene expression is important for macrophage survival and the innate immune response [33], [34]–[36].

Cholesterol is a multifunctional molecule that acts as an essential membrane component, a cofactor for signaling molecules and a precursor of steroid hormones [37], [38]. Although its role in the skin and skin appendages is incompletely understood, cholesterol is essential for epidermal barrier function [39] and forms an integral component of the hair follicle [40]. Several human malformation syndromes are caused by inherited enzyme defects in cholesterol biosynthesis and lead to a deficiency of cholesterol as well as increased levels of bioactive or toxic precursor sterols [41]. Three of these syndromes, CHILD, CDPX2 and desmosterolosis, exhibit multiple cutaneous abnormalities, including alopecia, and there are reports of scarring alopecia in patients with CDPX2 [41]. Recent studies by Evers et al [42] described hair growth defects in an Insig-deficient mouse model. These mice display defects in postnatal hair follicle cycling that result from the accumulation of cholesterol precursors; the defects could be rescued after treatment with simvastatin. Despite these reports, the role of cholesterol and other lipids in the pathogenesis of cutaneous disorders is not fully appreciated. Our study shows a direct link between the sterol precursors of cholesterol biosynthesis and hair disease in humans and may have implications for other skin and hair diseases that are immune-mediated, but whose mechanisms remain poorly defined.

This study reveals a previously unknown role for sterol intermediates of cholesterol biosynthesis in PCA pathogenesis and identifies a novel link between skin sterols and inflammation that may prove transformative in the diagnosis and treatment of these disorders. We conclude that the cholesterol biosynthetic pathway is impaired in the skin and hair follicles of PCA patients. Furthermore, investigations of PCA therapies should consider skin sterol levels as a potential therapeutic target and as a disease biomarker. A promising therapeutic strategy for PCA could be the restoration of cholesterol homeostasis in hair follicle cells by clinically available agonists of PPARγ or antagonists of LXR which are nuclear receptors that modulate this pathway [10], [43], [44].

## Materials and Methods

### Human Tissue

All research involving human subjects for this study has been conducted with written approval by the Case Institutional Review Board (IRB Protocol Number: 08-05-03). For research involving human participants, informed written consent has been obtained and all clinical investigation has been conducted according to the principles expressed in the Declaration of Helsinki. The diagnosis of PCA was based upon clinical observation and histopathologic findings of a lymphocytic, neutrophilic or mixed-cell infiltrate in the infundibular or the permanent portion of the hair follicles, as previously described [5]. The patients recruited for this study had early-active lesions that were judged to be clinically representative of lymphocytic (LPP, FFA, CCCA), neutrophilic (TF, FD) or mixed (DC) PCA. For microarray analysis, two 4-mm scalp biopsies were obtained, one from the affected area of the scalp and another from the clinically unaffected scalp. The affected biopsy specimens were obtained from the “active border,” which is an area with inflammation and retained (but decreased) hair follicles. These samples were paired with samples from clinically unaffected areas in the same patient. Scalp biopsy specimens from healthy volunteers (age-, sex- and race-matched) were included as controls. The normal controls examined had no evidence of hair or skin disorders. Scalp biopsies were obtained from patients seen at the clinics of the University Hospitals of Cleveland or at the University of California at San Francisco. All patients had active disease with itching, burning, pain, progressive hair loss, a positive pull test and evidence of inflammation. The patients were 18 years or older and were able to give informed consent, and the patients were evaluated in a standard manner that included a medical history; detailed hair questionnaire; treatment history; an examination of the hair, scalp, and skin and scalp photographs. All biopsies were performed under the approval of the Institutional Review Board and after appropriate consent had been obtained from patients and volunteers. All tissue samples were stored at −80°C until processed. These biopsies were utilized for total RNA extraction, microarray analysis and real-time PCR.

### Animals

All animal work has been conducted according to relevant national and international guidelines. All animal protocols for this study have been conducted with written approval by the Case Western Reserve University Institutional Animal Care and Use Committee (IACUC). Female C57BL/6J mice (Jackson Laboratory, Bar Harbor, Maine), aged 7 weeks, were randomly distributed into four groups. They were housed in groups of 5 with standard diet food pellets and water available *ad libitum*. Depilation to synchronize the hair cycle was performed as previously published [45], [46]. All of the hairs in the head region were removed by shaving, followed by treatment with a depilatory agent (Nair™ hair remover, Church and Dwight Co. Inc. Princeton, NJ, USA) to reveal pink skin. To compare the effects of different treatments on the hair cycle, the mice were treated topically with vehicle alone, with the sterol intermediate 7-DHC (Sigma-Aldrich, St. Louis, Missouri) or with the cholesterol biosynthesis inhibitor BM15766 (Sigma-Aldrich, St. Louis, Missouri) for 15 days. Each mouse was treated topically (painted every day on the head region) with the vehicle alone (ethanol: water 1∶1) or with 25 mM 7-DHC. The other animals were treated with vehicle alone (DMSO: water 1∶1) or with 4 mM BM15766. After 15 days of topical treatment, the mice were euthanized. Skin from the painted region was harvested and stored at −80°C for microarray and qRT-PCR analysis or embedded in paraffin for histological sectioning. The sections were stained with hematoxylin & eosin (H&E) using standard protocols for histological analysis.

### Human Hair Follicle Outer Root Sheath Cells (HHFORS cells)

HHFORS cells (ScienCell Research Laboratories, Carlsbad, CA) were grown in mesenchymal stem cell media (ScienCell Research Laboratories, Carlsbad, CA) with growth supplements (heat-inactivated FBS, penicillin, streptomycin and mesenchymal stem cell growth supplement) per the manufacturer’s instructions. Third- or fourth-passage cells were seeded at a density of 0.6×10^6^ cells per p100 plate. Ethanol was used to dissolve 7-DHC (Sigma-Aldrich, St. Louis, Missouri), and BM15766 (Sigma-Aldrich, St. Louis, Missouri) was dissolved in DMSO. The final ethanol and DMSO concentrations never exceeded 0.1% and did not affect the experiments. HHFORS cells in serum-free medium were treated with 7DHC, BM15766 or vehicle alone. A literature search revealed that the effects of 7-DHC and BM15766 had not been previously studied in HHFORS cells. We therefore conducted time- and concentration-varying treatments using the Vybrant® MTT Cell Proliferation Assay Kit (Invitrogen, Grand Island, NY; data not shown) to determine the optimal concentrations of 7-DHC (25 µM), BM15766 (4 µM) or vehicle alone and the optimal time of treatment (16–24 hours) for these experiments. Harvested cells were used for RNA isolation followed by microarray and RT-PCR analysis.

### RNA Isolation & Microarray Analysis

RNA isolation and microarray analysis were performed as described previously [10]. The total RNA from each biopsy was extracted using TRIzol reagent (Life Technologies, Inc., Gaithersburg, MD, USA) per the manufacturer's instructions. The RNA extraction was followed by purification using RNeasy Mini columns (Qiagen, Inc., Valencia, CA, USA). The RNA was quantified by spectrometry and used for microarray and real-time PCR experiments. The microarray experiments were performed with fresh frozen scalp tissue biopsies from patients with LPP (paired affected N = 6 & unaffected N = 5), CCCA (paired affected N = 3 and unaffected N = 3), FFA (paired affected N = 3 and unaffected N = 3) and TF (paired affected N = 4 and unaffected N = 3). These samples were compared to normal (control) scalp tissue (N = 10, pooled) by interrogating the Affymetrix GeneChip oligonucleotide array Human U133 Plus 2.0 (Affymetrix, Santa Clara, CA). Human hair follicle cells treated with 7-DHC and BM15766 were used for RNA isolation with the RNeasy Mini column and microarray analysis with the HG U133 Plus 2.0 Array.

The Mouse Gene 1.0 ST Array (Affymetrix, Santa Clara, CA) used for our studies provided whole-transcript coverage. Each of the 28,853 genes is represented on the array by approximately 27 probes spread across the full length of the gene to provide a more complete and accurate picture of gene expression than that possible with 3'-based expression array designs. Sense DNA targets were generated from as little as 100 ng of total RNA isolated from skin tissue painted with 7-DHC and BM15766. Mice treated with 7-DHC (N = 5) and BM15766 (N = 5) were compared with vehicle-treated controls.

Hybridization to the oligonucleotide arrays and subsequent washing and detection were performed as described in the Affymetrix Expression Analysis Technical Manual (Affymetrix). Array images were acquired using a GeneChip Scanner 3000 (Affymetrix), and the images were analyzed with the Genechip Operating Software (GCOS) and Expression Console. The image from each GeneChip was scaled such that the average intensity for all of the arrays was adjusted to a target intensity of 500 arbitrary units to account for the inherent differences between the chips and their hybridization efficiencies. The GeneChip array analysis yielded the hybridization intensity for each represented gene as the signal intensity. A multi-group Significance Analysis of Microarrays (SAM) approach [47] was undertaken to select a set of genes that was consistently differentially expressed among the unaffected and affected PCA and healthy scalp skin, with a false discovery rate (FDR) of <0.003. The difference between PCA and the normal controls was defined as significant for a 2-fold or greater difference in the average hybridization signal intensity with p<0.05 using a two-tailed unpaired *t-*test.

The Ingenuity Pathways Analysis (IPA) application (Ingenuity Systems, www.ingenuity.com) was used to identify functional significance, cellular location, and role in various biological and metabolic processes of the selected gene products. Details of the analytic methods and IPA data interpretation have been previously described [10]. Partek Genomics Suite (Partek Inc., St. Louis, Missouri) was used for hierarchical clustering and principal component analyses. Microarray data files from affected, unaffected and normal samples were imported into Partek for principal component analysis. All of the data sets were subjected to multi-way ANOVA, and transcripts with signals differing among groups with a certainty greater than p≤0.05 were selected for further study. A hierarchical cluster analysis was performed with Pearson’s dissimilarity and complete linkage.

### Quantitative Real-time RT-PCR

FAM-labeled PCR primers and TaqMan hydrolysis probes for all target genes and 18S rRNA or transferrin receptor (TFRC) controls were purchased from Applied Biosystems (Life Technologies Corporation, Carlsbad, California). Real-time PCR was performed on an ABI StepOne™ Sequence Detection System (Applied Biosystems, Carlsbad, California) according to the recommendations of the manufacturer. The expression of the target genes in all samples, including PCA (LPP, CCCA, FFA, DC, DF and TF) samples, control samples and samples from mice treated with 7-DHC, BM15766 or vehicle were quantified by the ΔΔC_T_ method, as described in the ABI StepOne™ Sequence Detection System manual (Applied Biosystems, Carlsbad, California).

### Histological Analysis

Mice treated with 7DHC and BM15766 were euthanized, and skin samples from the head region were harvested and placed in formalin overnight. Fixed skin samples were embedded in paraffin according to procedures used for routine histology and stained with H&E. For immunostaining of macrophages in PCA paraffin sections, CD68 mouse monoclonal antibodies (ab955; diluted 1∶100; Abcam, Cambridge, MA) were used. Macrophages in paraffin-embedded sections of mouse skin tissue were detected using rat monoclonal antibodies to F4/80 (ab6640; diluted 1∶100; Abcam, Cambridge, MA). Twenty-four-bit images were captured at 20× and 40× on an Olympus BX-60 upright microscope attached to a Retiga Exl Aqua camera (Q imaging Vancouver, BC).

### F4/80 Staining of Mouse Skin and Flow Cytometry

Cell suspensions were prepared from mouse skin that had been painted with the sterol intermediate 7-DHC or with the cholesterol inhibitor BM15766. Isolated cells were washed with RPM1/5% FBS and re-suspended in the same medium. The cells were counted and used for F4/80 flow cytometry. The cells were incubated with FC blocker (eBiosciences, San Diego, CA) for 20 min. After 20 minutes, the anti-mouse F4/80 antigen Alexa Fluor® 647 (eBioscience, San Diego, CA) or propidium iodide (BD Pharmingen San Diego, CA) was added. The samples were incubated for 30 minutes at 4°C with rotation. After 30 min, samples were washed with PBS twice and filtered with a 40-µm cell strainer. The filtered samples were analyzed with flow cytometry using the Accuri V6 flow cytometer (Ann Arbor, MI).

## Supporting Information

Figure S1 **Schematic representation of the cholesterol biosynthesis pathway.** Cholesterol biosynthesis involves the coordinated regulation of >20 enzymatic reactions. The early steps in endogenous cholesterol biosynthesis involve the conversion of acetyl-CoA to mevalonic acid via HMG-CoA. Statins inhibit the enzyme HMG-CoA reductase. The late steps involve the conversion of lathosterol to 7-dehydrocholesterol (7-DHC). The enzyme DHCR7 then converts 7-DHC to cholesterol. The cholesterol biosynthesis inhibitor BM15766 is a pharmacological inhibitor of the enzyme DHCR7.
(TIF)

Figure S2 **Real-time PCR validation of** ***TGFβ1and SOX9*** gene expression in HHFORS cells and in mouse skin (*p<0.05, **p<0.01) after treatment with 7-DHC and BM15766. Compared with untreated samples, *TGFβ1* gene expression was significantly increased both in HHFORS cells and in mouse skin after treatment with BM15766. No significant change in *TGFβ1* gene expression was observed after treatment with 7-DHC. In contrast, *SOX9* gene expression was significantly decreased both in HHFORS cells and in mouse skin after treatment with BM15766 and with 7-DHC. The unpaired *t-*test was used for statistical analysis.
(TIF)

Figure S3 **F4/80 staining of mouse tissue after treatment with 7-DHC and BM15766.** Macrophages in paraffin-embedded sections of mouse skin were detected using rat monoclonal antibodies to F4/80. Infiltration of macrophages was observed in mouse skin treated with 7-DHC and BM15766, but not in vehicle-treated controls (ethanol and DMSO). Twenty-four-bit images were captured at 20× and 40× on an Olympus BX-60 upright microscope attached to a Retiga Exl Aqua camera (Q Imaging, Vancouver, BC).
(TIF)

Figure S4 **CD68 staining of paraffin sections of normal scalp tissue and scalp tissue from patients with PCA (LPP, CCCA, FFA, and TF).** CD68+ cells were clearly observed in LPP, CCCA and TF; fewer CD68+ cells were observed in FFA. Twenty-four-bit images were captured at 40× on an Olympus BX-60 upright microscope attached to a Retiga Exl Aqua camera (Q Imaging, Vancouver, BC).
(TIF)

Table S1 **The expression of cholesterol biosynthesis genes is decreased in PCA.** The degree of change of 14 differentially expressed cholesterol biosynthesis genes in LPP, CCCA, FFA and TF is shown (N = 10 for each subtype). Although cholesterol biosynthesis genes are downregulated in all subtypes of PCA, different sets of genes are downregulated in different subtypes.
(TIF)

Table S2 **Interferon response genes in PCA.** The expression of interferon signaling genes in LPP, CCCA, FFA and TF is shown. The expression of these genes in samples from patients with PCA is compared with that in normal tissue. Interferon response genes are significantly upregulated in LPP. Fewer interferon-responsive genes are differentially expressed in the other PCA subtypes. (ND =  No change detected.)
(TIF)

Table S3 **Expression of genes in the** ***TLR4*** **and** ***TLR6*** **networks.** The *TLR4*- and *TLR6*-predicted networks were generated using IPA. The identity of the genes and the degree of change in the *TLR4* network (Figure 5C) induced by treatment of HHFORS cells with 7-DHC and in the *TLR6* network (Figure 5D) after treatment of HHFORS cells with BM15766 are shown.
(TIF)

Table S4 **Expression of genes in the** ***TLR*** **and** ***IFN*** **networks that are activated upon treatment of mouse skin with 7-DHC.** The identity of the genes and the degree of changes in the *TLR* and *IFN* networks that are activated after treatment of mouse skin with 7-DHC are shown. Several inflammatory genes, including interferon responsive genes, are upregulated in both networks.
(TIF)

Glossary S1 **A list of all abbreviations, gene symbols and gene names included in the manuscript are shown in the glossary.**
(PDF)
