# Supplementary figures and images for: Sterol Intermediates of Cholesterol Biosynthesis Inhibit Hair Growth and Trigger an Innate Immune Response in Cicatricial Alopecia
Source: PLoS One. 2012 Jun 7;7(6):e38449. doi: 10.1371/journal.pone.0038449 (PMC3369908; doi:10.1371/journal.pone.0038449)

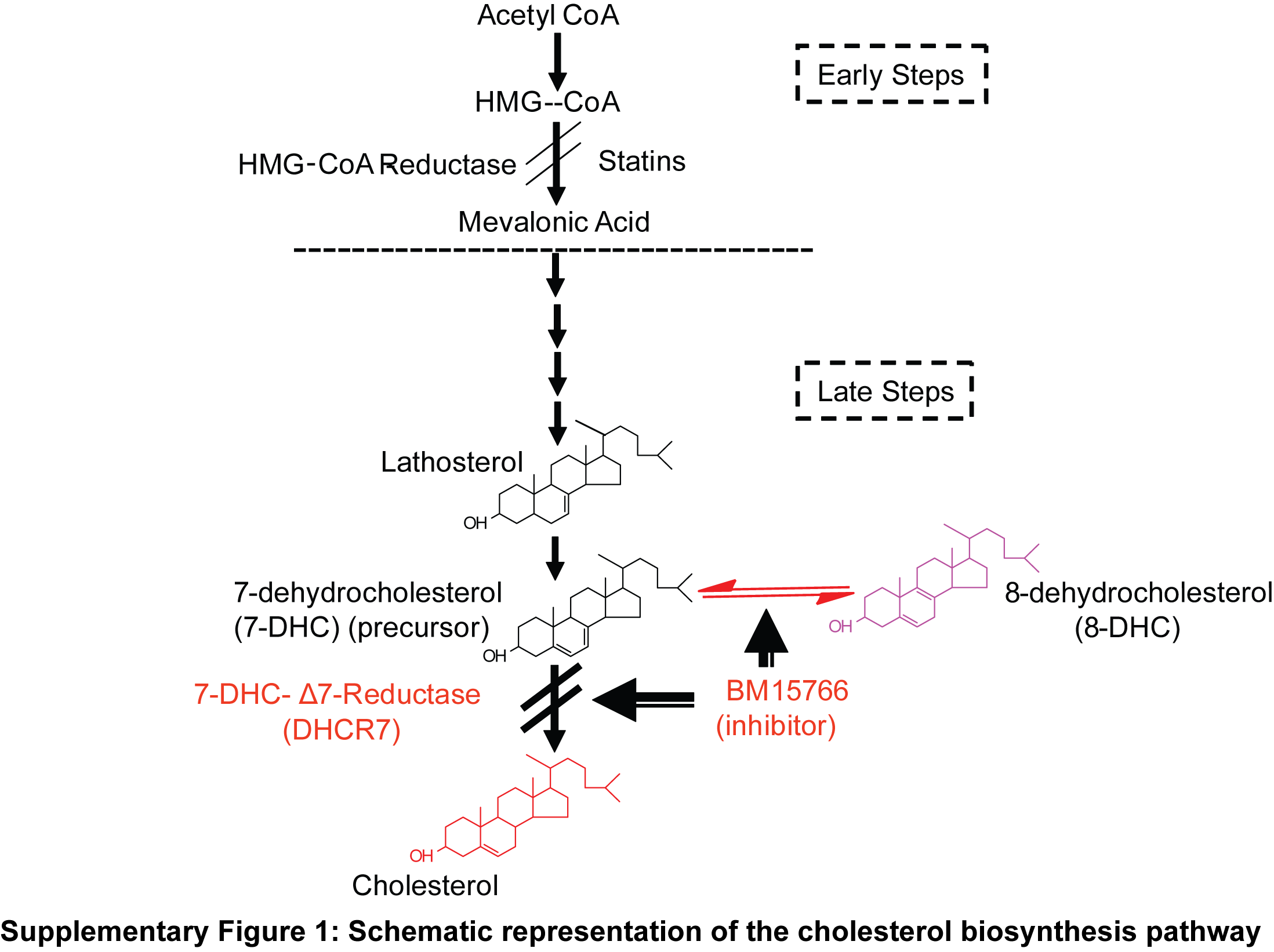

Supplement: Figure S1 — Schematic representation of the cholesterol biosynthesis pathway. Cholesterol biosynthesis involves the coordinated regulation of >20 enzymatic reactions. The early steps in endogenous cholesterol biosynthesis involve the conversion of acetyl-CoA to mevalonic acid via HMG-CoA. Statins inhibit the enzyme HMG-CoA reductase. The late steps involve the conversion of lathosterol to 7-dehydrocholesterol (7-DHC). The enzyme DHCR7 then converts 7-DHC to cholesterol. The cholesterol biosynthesis inhibitor BM15766 is a pharmacological inhibitor of the enzyme DHCR7. (TIF) [file pone.0038449.s001.tif]

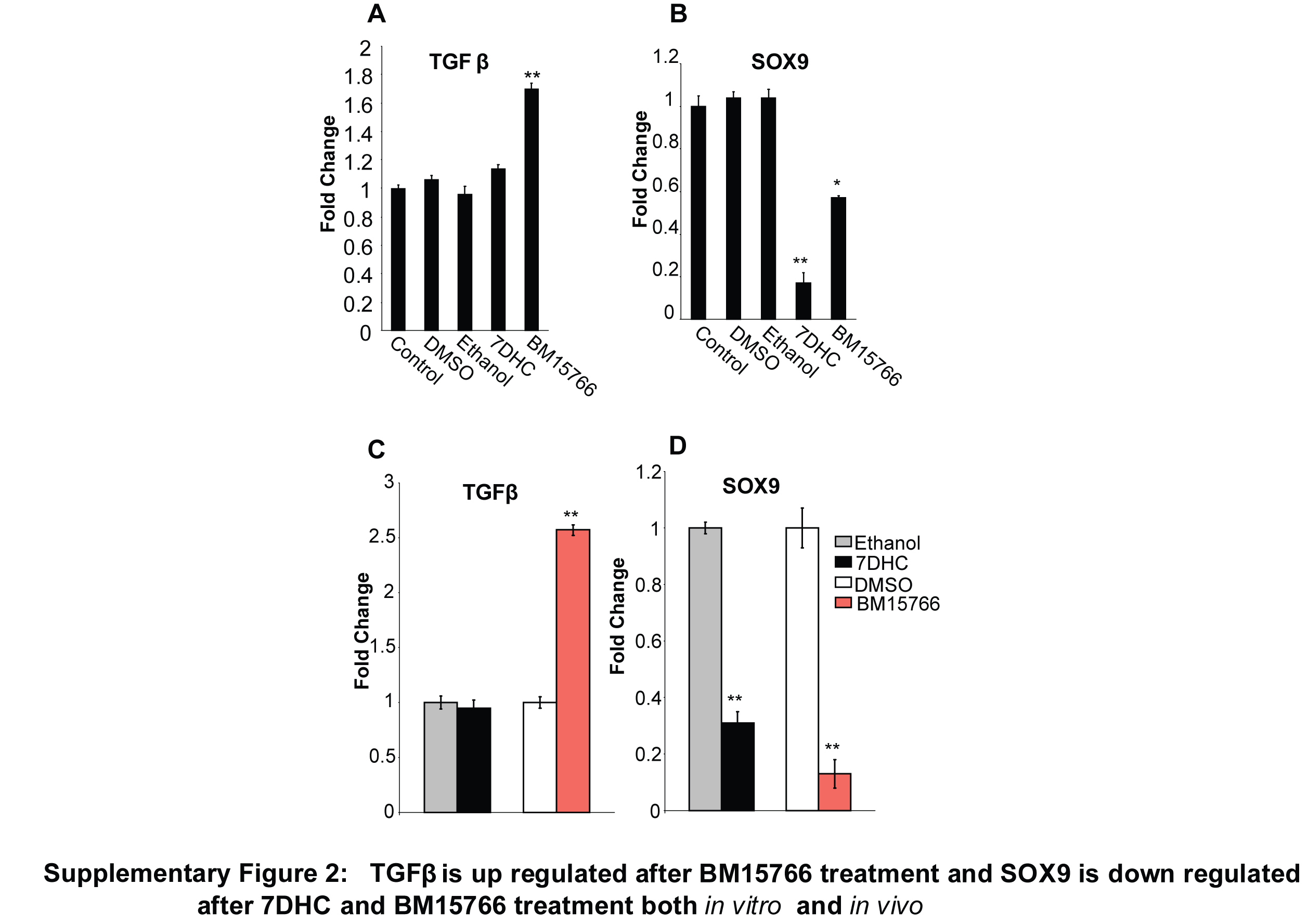

Supplement: Figure S2 — Real-time PCR validation of TGFβ1and SOX9 gene expression in HHFORS cells and in mouse skin (*p<0.05, **p<0.01) after treatment with 7-DHC and BM15766. Compared with untreated samples, TGFβ1 gene expression was significantly increased both in HHFORS cells and in mouse skin after treatment with BM15766. No significant change in TGFβ1 gene expression was observed after treatment with 7-DHC. In contrast, SOX9 gene expression was significantly decreased both in HHFORS cells and in mouse skin after treatment with BM15766 and with 7-DHC. The unpaired t-test was used for statistical analysis. (TIF) [file pone.0038449.s002.tif]

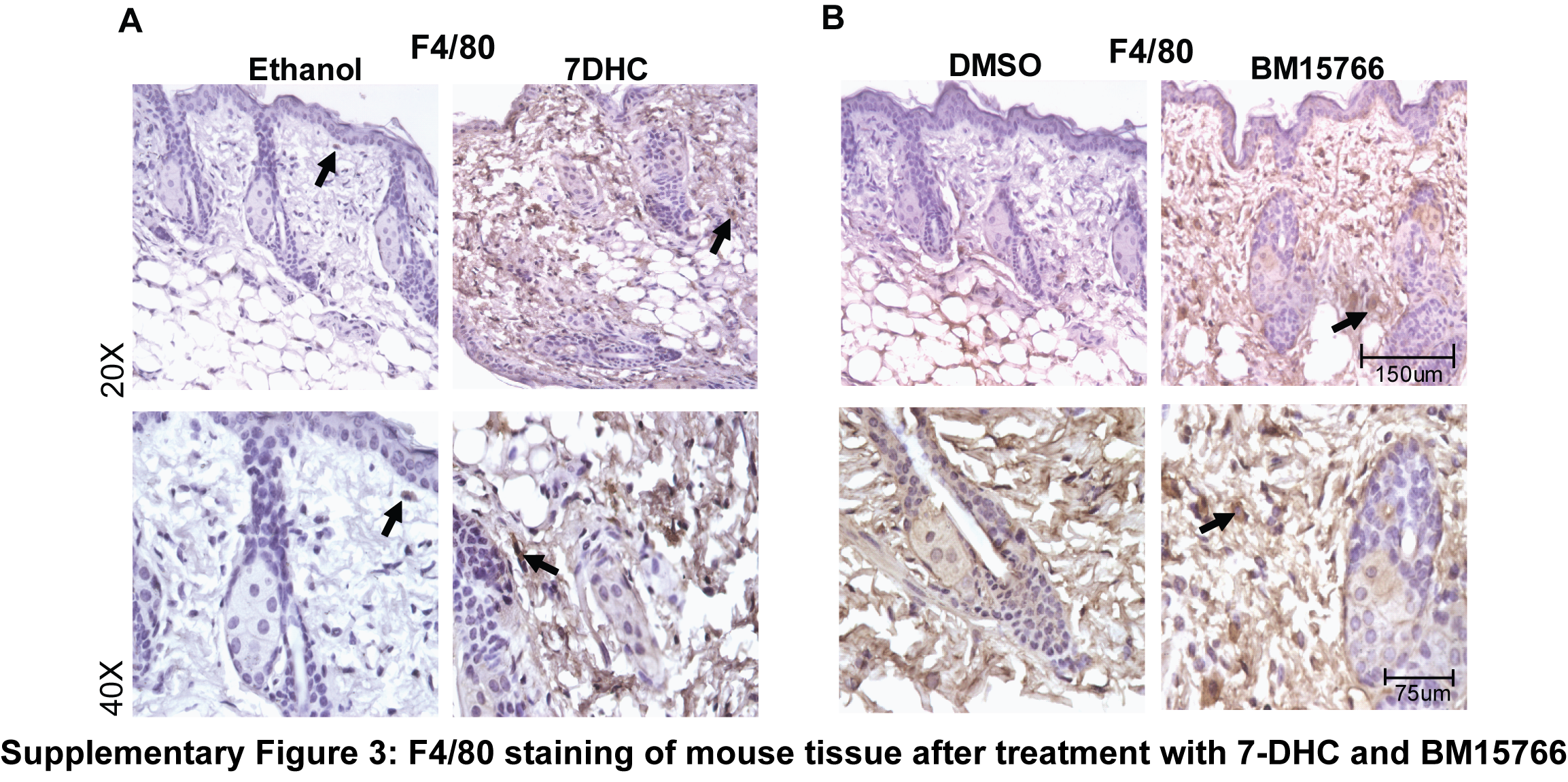

Supplement: Figure S3 — F4/80 staining of mouse tissue after treatment with 7-DHC and BM15766. Macrophages in paraffin-embedded sections of mouse skin were detected using rat monoclonal antibodies to F4/80. Infiltration of macrophages was observed in mouse skin treated with 7-DHC and BM15766, but not in vehicle-treated controls (ethanol and DMSO). Twenty-four-bit images were captured at 20× and 40× on an Olympus BX-60 upright microscope attached to a Retiga Exl Aqua camera (Q Imaging, Vancouver, BC). (TIF) [file pone.0038449.s003.tif]

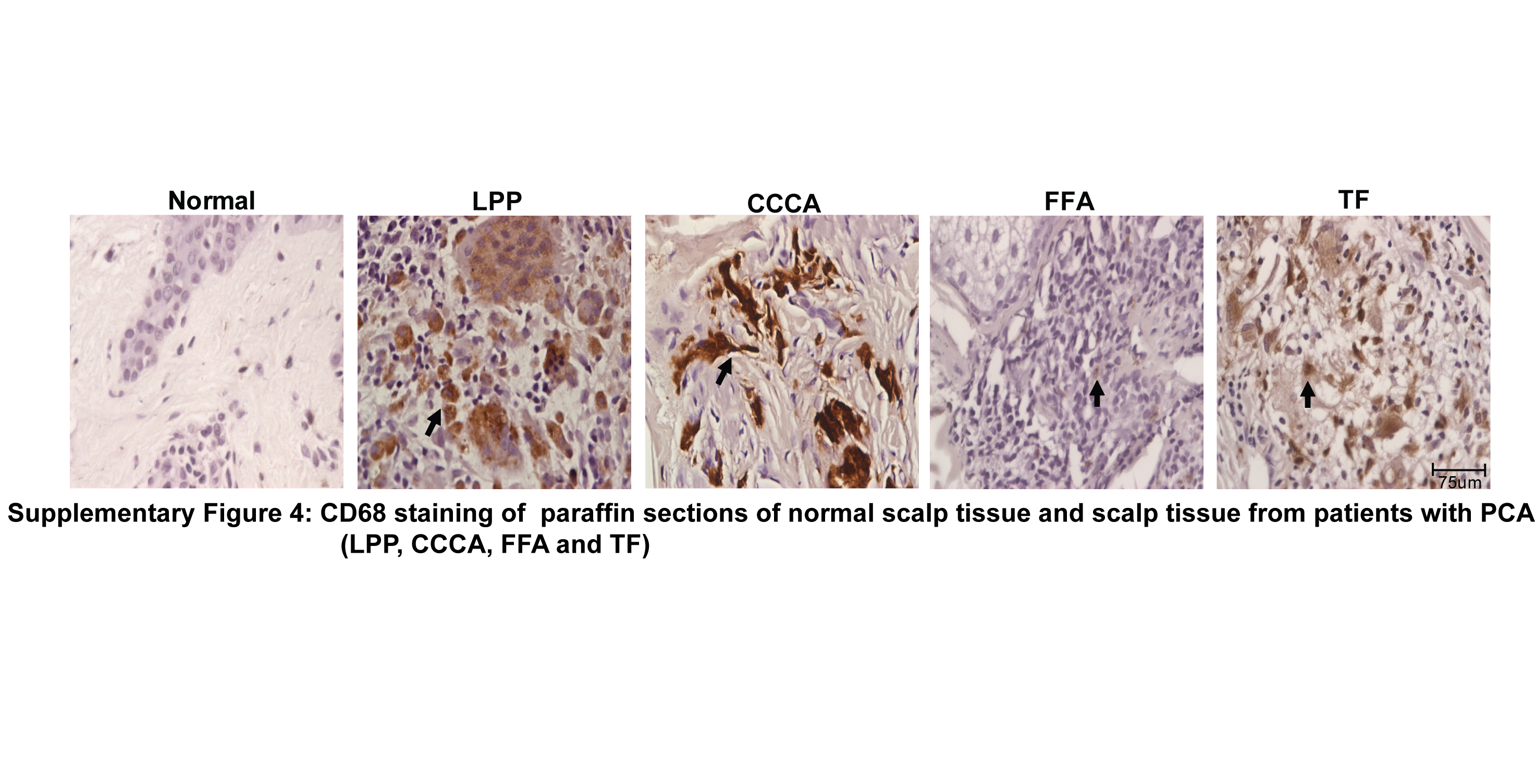

Supplement: Figure S4 — CD68 staining of paraffin sections of normal scalp tissue and scalp tissue from patients with PCA (LPP, CCCA, FFA, and TF). CD68+ cells were clearly observed in LPP, CCCA and TF; fewer CD68+ cells were observed in FFA. Twenty-four-bit images were captured at 40× on an Olympus BX-60 upright microscope attached to a Retiga Exl Aqua camera (Q Imaging, Vancouver, BC). (TIF) [file pone.0038449.s004.tif]

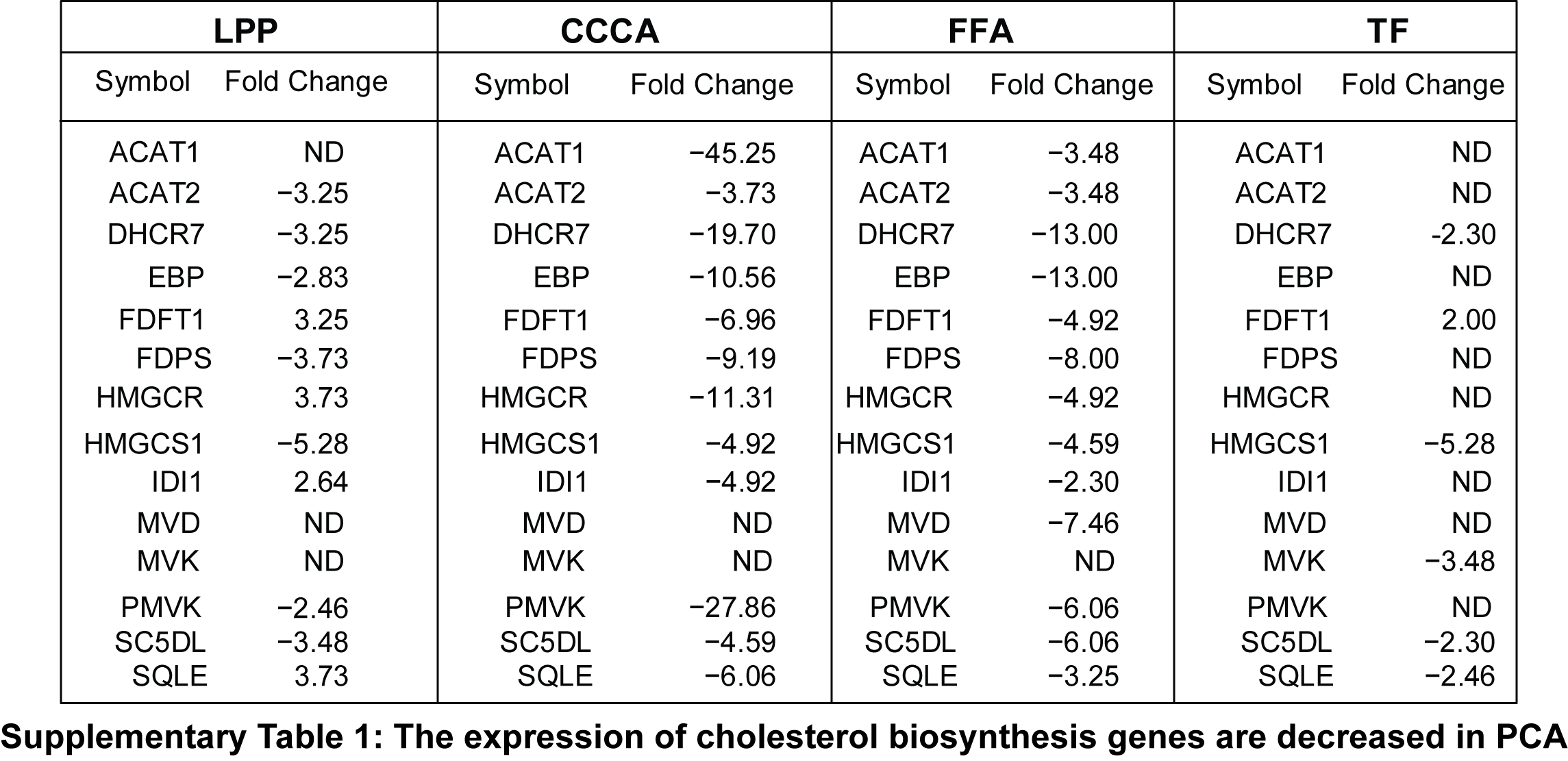

Supplement: Table S1 — The expression of cholesterol biosynthesis genes is decreased in PCA. The degree of change of 14 differentially expressed cholesterol biosynthesis genes in LPP, CCCA, FFA and TF is shown (N = 10 for each subtype). Although cholesterol biosynthesis genes are downregulated in all subtypes of PCA, different sets of genes are downregulated in different subtypes. (TIF) [file pone.0038449.s005.tif]

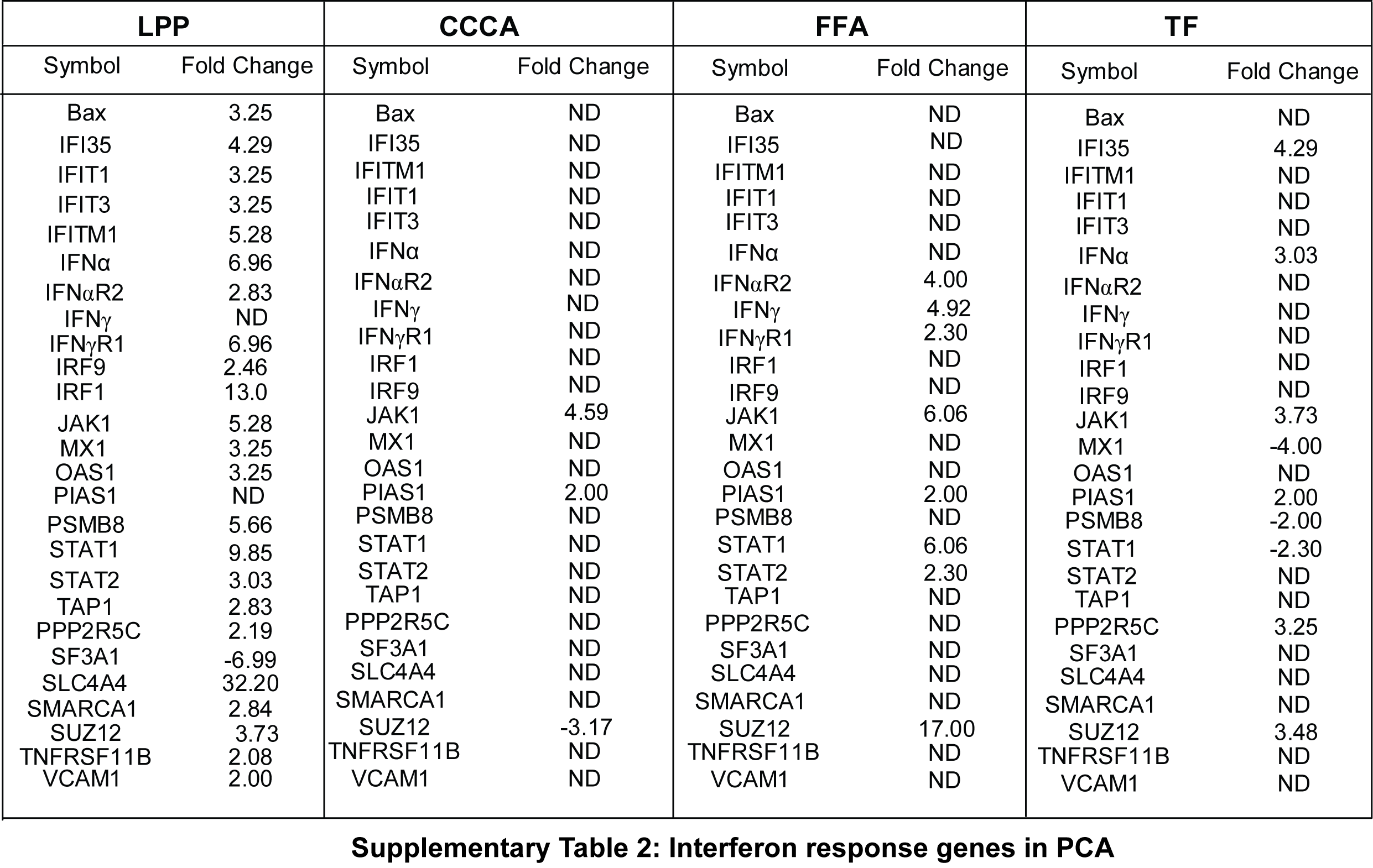

Supplement: Table S2 — Interferon response genes in PCA. The expression of interferon signaling genes in LPP, CCCA, FFA and TF is shown. The expression of these genes in samples from patients with PCA is compared with that in normal tissue. Interferon response genes are significantly upregulated in LPP. Fewer interferon-responsive genes are differentially expressed in the other PCA subtypes. (ND = No change detected.) (TIF) [file pone.0038449.s006.tif]

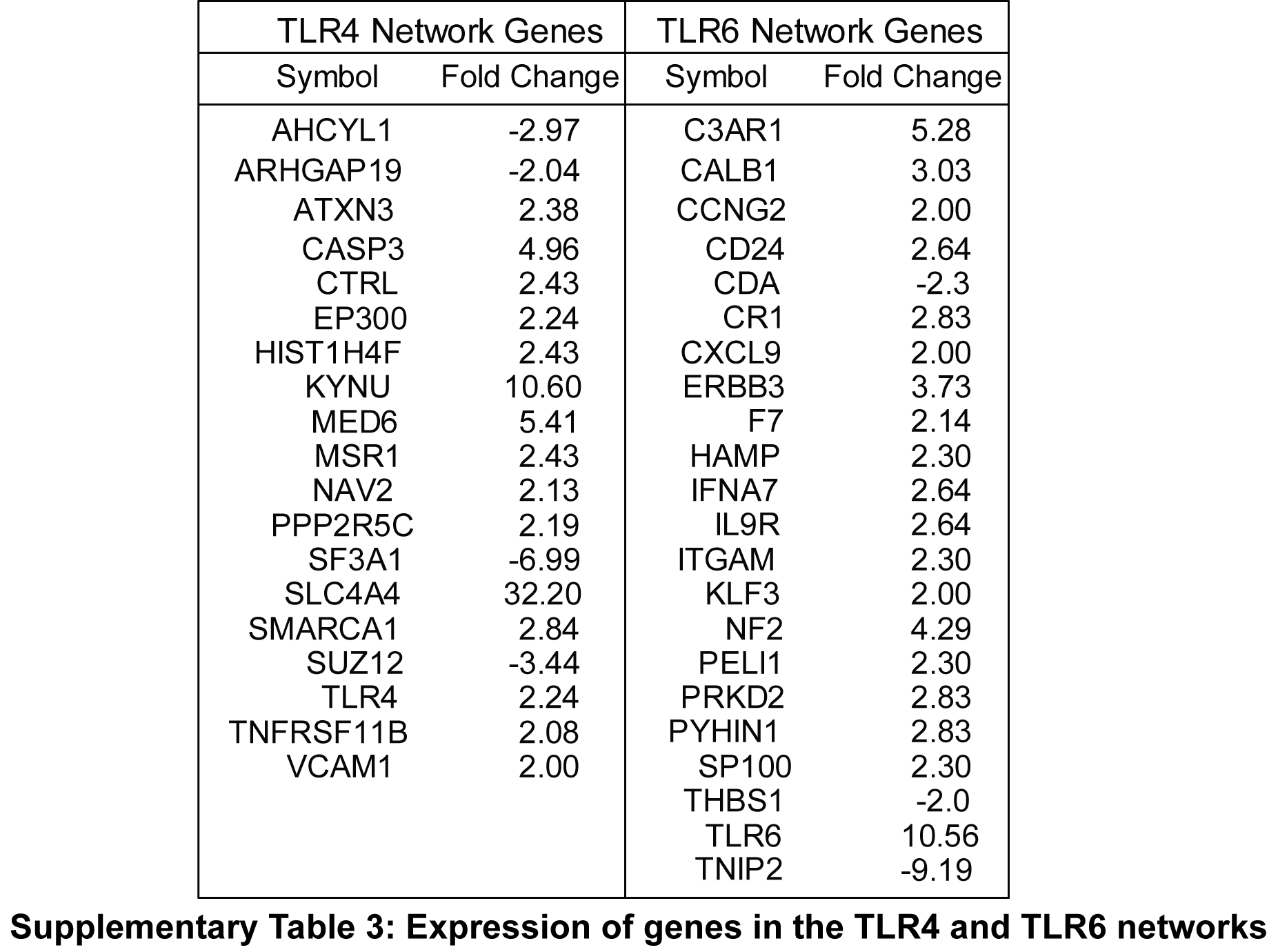

Supplement: Table S3 — Expression of genes in the TLR4 and TLR6 networks. The TLR4- and TLR6-predicted networks were generated using IPA. The identity of the genes and the degree of change in the TLR4 network (Figure 5C) induced by treatment of HHFORS cells with 7-DHC and in the TLR6 network (Figure 5D) after treatment of HHFORS cells with BM15766 are shown. (TIF) [file pone.0038449.s007.tif]

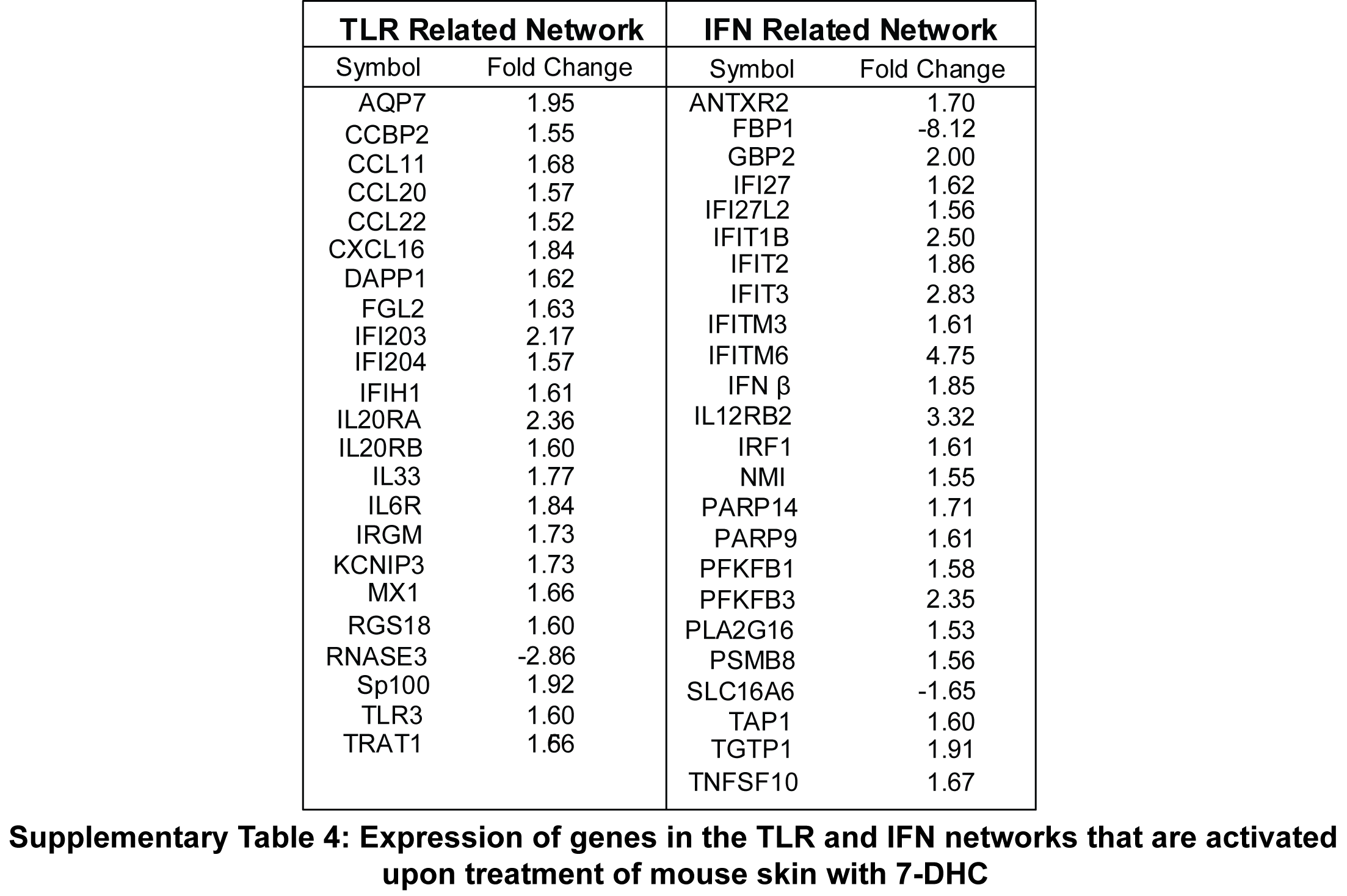

Supplement: Table S4 — Expression of genes in the TLR and IFN networks that are activated upon treatment of mouse skin with 7-DHC. The identity of the genes and the degree of changes in the TLR and IFN networks that are activated after treatment of mouse skin with 7-DHC are shown. Several inflammatory genes, including interferon responsive genes, are upregulated in both networks. (TIF) [file pone.0038449.s008.tif]
