# Supplementary material for: Sterol Intermediates of Cholesterol Biosynthesis Inhibit Hair Growth and Trigger an Innate Immune Response in Cicatricial Alopecia
Source: PLoS One. 2012 Jun 7;7(6):e38449. doi: 10.1371/journal.pone.0038449 (PMC3369908; doi:10.1371/journal.pone.0038449)
Supplement: Glossary S1 — A list of all abbreviations, gene symbols and gene names included in the manuscript are shown in the glossary. (PDF) [file pone.0038449.s009.pdf]

Glossary

LPP	Lichen Planopilaris
CCCA	Central Centrifugal Cicatricial Alopecia
FFA	Frontal Fibrosis alopecia
TF	Tufted folliculitis
DC	Dissecting cellulitis
DHCR7	7-dehydrocholesterol reductase
EBP	Emopamil-binding protein
TLR3	Toll-like receptor-3
TLR4	Toll-like receptor-4
TLR6	Toll-like receptor-6
IFNα	Interferon alpha
IFNα7	Interferon alpha7
NFκB	Nuclear factor kappa B
MCP1	Monocyte chemotactic protein-1
7DHC	7-Dehydrocholesterol
BM 15766	BM 15766 sulfate
HF	Hair Follicle
PPARγ	Peroxisome proliferator-actvated receptor gamma
SC5DL	sterol-C5-desaturase
FDPS	farnesyl diphosphate synthase
MVK	mevalonate kinase
MVD	mevalonate (diphospho) decarboxylase
NK Cell	natural killer cell
IL	interleukin
MAP kinase	mitogen-activated protein kinase
SLE	Systemic lupus erythematosus
CHILD Syndrome	Congenital hemidysplasia with ichthyosiform erythroderma and limb defects
CDPX2	Conradi–Hünermann–Happle syndrome
HHFORS	Human hair follicle outer root sheath cells

Glossary

ACAT1	acetyl-CoA acetyltransferase 1
ACAT2	acetyl-CoA acetyltransferase 2
FDFT1	farnesyl-diphosphate farnesyltransferase 1
FDPS	farnesyl diphosphate synthase
HMGCS1	3-hydroxy-3-methylglutaryl-CoA synthase 1
HMGCR	3-hydroxy-3-methylglutaryl-CoA reductase
IDI1	isopentenyl-diphosphate delta isomerase 1
SC5DL	sterol-C5-desaturase
SQLE	squalene epoxidase
MVD	mevalonate (diphospho) decarboxylase
MVK	mevalonate kinase
DHCR7	7-dehydrocholesterol reductase
EBP	Emopamil-binding protein
PMVK	phosphomevalonate kinase
BAX	BCL2-associated X protein
IFI35	interferon-induced protein 35
IFIT1	interferon-induced protein with tetratricopeptide repeats 1
IFIT3	interferon-induced protein with tetratricopeptide repeats 3
IFITM1	interferon induced transmembrane protein 1
IFNα	Interferons alpha
IFNαR2	Interferons alpha Receptor 2
IFNγ	interferon *gamma*
IFNγR2	interferon *gamma* receptor 2
IRF9	interferon regulatory factor 9
IRF1	interferon regulatory factor 1
JAK1	Janus kinase 1
MX1	myxovirus resistance 1

Glossary

OAS1	2'-5'-oligoadenylate synthetase 1
PIAS1	protein inhibitor of activated STAT, 1
PSMB8	proteasome subunit, beta type, 8
Stat1	signal transducer and activator of transcription 1
Stat2	signal transducer and activator of transcription 2
TAP1	transporter 1, ATP-binding cassette
PPP2R5C	protein phosphatase 2, regulatory subunit B', gamma
SF3A1	splicing factor 3a, subunit 1
SLC4A4	Solute carrier family 4, sodium bicarbonate cotransporter, member 4
SUZ12	suppressor of zeste 12 homolog
TNFRSF11B	tumor necrosis factor receptor superfamily, member 11b
VCAM1	vascular cell adhesion molecule 1
AHCYL1	adenosylhomocysteinase-like 1
ARHGAP19	Rho GTPase activating protein 19
ATXN3	ataxin 3
CASP3	caspase 3, apoptosis-related cysteine peptidase
CTRL	chymotrypsin-like
EP300	E1A binding protein p300
HIST1H4F	histone cluster 1, H4f
KYNU	kynureninase
MED6	mediator complex subunit 6
MSR1	macrophage scavenger receptor 1
NAV2	neuron navigator 2
SMARCA1	SWI/SNF related, matrix associated, actin dependent regulator of chromatin, subfamily a, member 1
C3AR1	complement component 3a receptor 1
CALB1	calbindin 1, 28kDa
CCNG2	cyclin G2
CDA	cytidine deaminase
CR1	complement component receptor 1
CXCL9	chemokine (C-X-C motif) ligand 9
ERBB3	v-erb-b2 erythroblastic leukemia viral oncogene homolog 3 (avian)
F7	coagulation factor VII

Glossary

HAMP	hepcidin antimicrobial peptide
IL9R	interleukin 9 receptor
ITGAM	integrin, alpha M (complement component 3 receptor 3 subunit)
KLF3	Kruppel-like factor 3 (basic)
NF2	neurofibromin 2
PELI1	pellino homolog 1
PRKD2	protein kinase D2
PYHIN1	pyrin and HIN domain family, member 1
SP100	SP100 nuclear antigen
THBS1	thrombospondin 1
TNIP2	TNFAIP3 interacting protein
AQP7	aquaporin 7
CCBP2	chemokine binding protein 2
CCL11	chemokine (C-C motif) ligand 11
CCL20	chemokine (C-C motif) ligand 20
CCL22	chemokine (C-C motif) ligand 22
CXCL16	chemokine (C-X-C motif) ligand 16
DAPP1	dual adaptor of phosphotyrosine and 3-phosphoinositides
FGL2	fibrinogen-like 2
Ifi203	interferon activated gene 203
Ifi204	interferon activated gene 204
IFIH1	interferon induced with helicase C domain 1
IL20RA	interleukin 20 receptor beta
IL20RB	interleukin 20 receptor beta
IL33	interleukin 33
IL6R	interleukin 6 receptor
IRGM	immunity-related GTPase family, M
KCNIP3	Kv channel interacting protein 3, calsenilin
RGS18	regulator of G-protein signaling 18
RNASE3	ribonuclease, RNase A family, 3
TLR3	toll-like receptor 3

Glossary

TRAT1	T cell receptor associated transmembrane adaptor 1
ANTXR212	anthrax toxin receptor 2
FBP1	fructose-1,6-bisphosphatase 1
GBP2	guanylate binding protein 2, interferon-inducible
IFI27	interferon, alpha-inducible protein 27
IFI27L2	interferon, alpha-inducible protein 27-like 2
IFIT1B	interferon-induced protein with tetratricopeptide repeats 1B
IFIT2	interferon-induced protein with tetratricopeptide repeats 2
IFIT3	interferon-induced protein with tetratricopeptide repeats 3
IFITM3	interferon induced transmembrane protein 3 (1-8U)
Ifitm6	interferon induced transmembrane protein 6
Iigp1b	interferon inducible GTPase 1
IL12RB2	interleukin 12 receptor, beta 2
IRF1	interferon regulatory factor 1
NMI	N-myc (and STAT) interactor
PARP14	poly (ADP-ribose) polymerase family, member 14
PARP9	poly (ADP-ribose) polymerase family, member 9
PFKFB1	6-phosphofructo-2-kinase/fructose-2,6-biphosphatase 1
PFKFB3	6-phosphofructo-2-kinase/fructose-2,6-biphosphatase 3
PLA2G16	phospholipase A2, group 16
PSMB8	proteasome (prosome, macropain) subunit, beta type, 8 (large multifunctional peptidase 7)
SLC16A6	solute carrier family 16, member 6 (monocarboxylic acid transporter 7)
TAP1	transporter 1, ATP-binding cassette, sub-family B (MDR/TAP)
Tgtp1	T-cell specific GTPase 1
TNFSF10	tumor necrosis factor (ligand) superfamily, member 10
TGF-β	Transforming growth factor *beta*
SOX9	SRY (sex determining region Y)-box 9
